# Somnambulism: Emergency Department Admissions Due to Sleepwalking-Related Trauma

**DOI:** 10.5811/westjem.2016.8.31123

**Published:** 2016-09-29

**Authors:** Thomas C. Sauter, Sajitha Veerakatty, Dominik G. Haider, Thomas Geiser, Meret E. Ricklin, Aristomenis K. Exadaktylos

**Affiliations:** * University Hospital Berne, Department of Emergency Medicine, Inselspital, Bern, Switzerland; † University Hospital Berne, Department of Pulmonary Medicine, Inselspital, Bern, Switzerland

## Abstract

**Introduction:**

Somnambulism is a state of dissociated consciousness, in which the affected person is partially asleep and partially awake. There is pervasive public opinion that sleepwalkers are protected from hurting themselves. There have been few scientific reports of trauma associated with somnambulism and no published investigations on the epidemiology or trauma patterns associated with somnambulism.

**Methods:**

We included all emergency department (ED) admissions to University Hospital Inselspital, Berne, Switzerland, from January 1, 2000, until August 11, 2015, when the patient had suffered a trauma associated with somnambulism. Demographic data (age, gender, nationality) and medical data (mechanism of injury, final diagnosis, hospital admission, mortality and medication on admission) were included.

**Results:**

Of 620,000 screened ED admissions, 11 were associated with trauma and sleepwalking. Two patients (18.2%) had a history of known non-rapid eye movement parasomnias. The leading cause of admission was falls. Four patients required hospital admission for orthopedic injuries needing further diagnostic testing and treatment (36.4%). These included two patients with multiple injuries (18.2%). None of the admitted patients died.

**Conclusion:**

Although sleepwalking seems benign in the majority of cases and most of the few injured patients did not require hospitalization, major injuries are possible. When patients present with falls of unknown origin, the possibility should be evaluated that they were caused by somnambulism.

## INTRODUCTION

Sleepwalking (somnambulism) is a state of dissociated consciousness, in which the affected person is partially asleep and partially awake.^1^,^2^ There is pervasive public opinion that sleepwalkers are protected from hurting themselves. There have been few scientific reports of trauma associated with somnambulism,^3^,^4^ and there are no published investigations on the epidemiology or trauma patterns associated with somnambulism.

Somnambulism typically occurs at the transition from deep non-rapid eye movement (NREM) sleep before it progresses to REM sleep.^1^ It is therefore regarded as a disorder of impaired arousal. Most deep NREM sleep is in the first third of the night, so that the somnambulistic event usually occurs one to three hours after sleep onset, but ends when arousal is complete and full wakefulness is reached. It is generally followed by a rapid return to sleep. The patient exhibits complete amnesia of the episode upon awakening.^1^,^5^,^6^ He may notice changes in the household (overturned furniture, flowerpots) or have personal injuries (scratches, wounds) after the episode.

In the average population, about 2–3% of adults sleepwalk.^7^ Disorders of arousal are especially common in childhood; 15% of children aged 2.5 to 6 years are estimated to have sleepwalked at least once, in comparison with 6% of children aged 6 to 11 years. The prevalence decreases significantly with age, because slow-wave sleep is most abundant in children.^1^,^8^,^9^ However, all factors that increase the amount of deep sleep (drugs [especially psychotropics], sleep deprivation, stress, restless legs syndrome, sleep disordered breathing, thyrotoxicosis or pregnancy) can provoke a parasomniac episode.^10^,^11^

Our study aims to provide insight into the type of injuries that may be encountered associated with sleepwalking and provide demographic information about patients with emergency department (ED) admissions due to sleepwalking-related trauma.

## METHODS

For this retrospective chart review, we screened the computerized database (E-Care, ED 2.1.3.0, Turnhout, Belgium) of all ED admissions to University Hospital Inselspital, Bern, Switzerland, from January 1, 2000, until August 11, 2015, (n=620,000) for trauma cases associated with somnambulism (key words “Schlafwandler,” “schlafwandeln” [English: “sleepwalker” and “sleepwalking”). A catchment area of about two million people is covered by the ED of University Hospital Berne, a Level I trauma center for adults > 15 years. The ED is a self-contained, interdisciplinary unit and treats approximately 500 multi-injured patients (Injury-Severity-Index >16) per year; about 40% of patients are admitted for surgical reasons and about 30% of patients are admitted to the hospital.^12^ Demographic data (age, gender, nationality) and medical data (mechanism of injury, final diagnosis, hospital admission, mortality and medication on admission) were extracted from the patient records, anonymized, double-checked for documentation errors and included in our investigation.

## RESULTS

Out of 620,000 ED admissions, 11 trauma admissions were associated with a reported history of sleepwalking. For characteristics and injury patterns see the table.

The mean age was 39 years (range, 16 to 77 years); four patients were female and seven were male (36.4% vs. 63.6%). The leading cause of admission was a fall (mostly from bed, stairs or windows). Two patients (18.2%) had a history of known NREM parasomnias. The most common co-morbidity was epilepsy (n=6, 54.5%). None of the patients was on psychotropic drugs at admission. Seven patients (63.6%) were managed in the outpatient setting. Four patients (36.4%) required hospital admission because of orthopedic injuries needing further diagnostic testing and treatment. These included two patients with multiple injuries (18.2%). None of the admitted patients died.

## DISCUSSION

Trauma associated with somnambulism is rare but may be potentially life threatening.

Previously identified factors that initiate sleepwalking include drugs and psychotropic medications.^10^ In one of the earliest reports (1979), Charney et al. showed an association between use of neuroleptic medication and sleepwalking.^13^ There are other published reports that psychotropic medications can induce a somnambulistic episode.^4^,^14^,^15^ None of our trauma patients was on psychotropic medications at admission. However, given the potential risk of triggering sleepwalking episodes, psychotropic medications should be used with caution in susceptible patients.

Although 2–3% of adults do sleepwalk,^7^ the very low number (n=11) of sleepwalking-associated accidents out of about 620,000 ED admissions seems to confirm the pervasive public opinion that sleepwalkers are protected from hurting themselves. Nevertheless, our study shows that life-threatening injuries associated with somnambulism may occur.

The rare incidence of trauma in sleepwalkers combined with emergency physicians’ lack of awareness of the danger of somnambulism may cause the diagnosis of somnambulism to be missed in patients presenting with falls of unknown origin. Obtaining a detailed history from the patient as well as from the family may be the only possibility to establish the diagnosis of sleepwalking. In our patients, only a small minority of patients had a known previous history of sleepwalking. Under-diagnosed cases of somnambulism, together with medications that potentially initiate sleepwalking, may lead to preventable injuries. In patients with known sleepwalking, avoidance of sleep deprivation and medications or substances associated with disorders of arousal like alcohol, psychotropic and hypnotic medications is essential.^10^ Additionally, given the limited treatment options of sleepwalking, it is necessary to educate susceptible patients about risk-mitigation strategies to prevent injuries.

## LIMITATION

Our study is limited to patients older than 15 years because children are not treated in our ED. Given the higher prevalence of somnambulism in younger children than in adults, evaluation of sleepwalking accidents in a pediatric population would be important.

As with all retrospective studies involving medical records, there is no guarantee that all patients in the large database were found and correctly reported. Another limitation to our study is the small number of sleepwalking-associated trauma cases that could be reported despite screening of a large patient population. Our investigation aims to promote awareness to the topic of sleepwalking-associated injuries and might stimulate further research on a topic that has not been extensively studied so far. Prospective investigations of patients with somnambulism and trauma should be conducted to identify further, potentially preventable, risk factors.

## CONCLUSION

Although sleepwalking seems benign in the majority of cases and most of the few injured patients will not require hospitalization, major injuries are possible. Injuries related to sleepwalking may be potentially overlooked and a high index of suspicion is important. When patients present with falls of unknown origin, the possibility of a somnambulistic cause should be considered. In patients at risk of sleepwalking, the use of psychotropic medications has to be closely evaluated.

## Figures and Tables

**Table:** Patient characteristics and traumatic injuries associated with somnambulism.

Age	Gender	Outpatient setting	Injuries
63	M	Yes	Multiple contused facial lacerations
77	F	Yes	Thoracic haematoma
38	M	No	Severe head injury, serious craniofacial injuries, third degree open fracture of the femur, cervical spinal dislocation at the fifth and sixth cervical vertebral bodies, soft tissue injuries
26	F	Yes	Contusion of the ribs and right knee
43	M	No	Intra-articular distal radius fracture, contused laceration facial
59	F	No	Cerebral concussion, luxation fracture of the facet joint of the sixth and seventh cervical vertebral bodies with neuroforaminal stenosis and epidural haematoma, fracture of the skull (parietal left and temporal right) and of the left lateral orbital wall, exophthalmos left.
23	M	Yes	Minor head injury, temporoparietal excoriation, fracture of the left clavicle
16	M	Yes	Upper back contusion
16	F	Yes	Contusion of the right ankle joint and sternum, superficial cuts on left lower leg
20	M	Yes	Shoulder luxation on the left side
53	M	No	Paraplegia below thoracic vertebral body 10, pneumothorax on the left side, aspiration on the right side

* n=11
